# Paired comparisons of mutational profiles before and after brachytherapy in asian uveal melanoma patients

**DOI:** 10.1038/s41598-021-98084-8

**Published:** 2021-09-20

**Authors:** Woo Seung Lee, Junwon Lee, Jun Jeong Choi, Hyun Goo Kang, Sung Chul Lee, Ju Han Kim

**Affiliations:** 1 Division of Biomedical Informatics, Seoul National University Biomedical Informatics (SNUBI), Seoul National University College of Medicine, Seoul, 03080 South Korea; 2 Department of Ophthalmology, Institute of Human Barrier Research, Gangnam Severance Hospital, Yonsei University College of Medicine, Seoul, South Korea; 3 Department of Pharmacy and Yonsei Institute of Pharmaceutical Sciences, College of Pharmacy, Yonsei University, Incheon, 06273 South Korea; 4 Department of Ophthalmology, Konyang University College of Medicine, Daejeon, South Korea

**Keywords:** Eye cancer, Cancer genomics, Eye cancer

## Abstract

Uveal melanoma(UM) is the most common primary intraocular malignancy in adults. However, the incidence of UM in Asia is 10 to 20 times less than in Western populations. Therefore, for the first time, we report our whole exome sequencing (WES) data analysis to discover differences in the molecular features of Asian and Western UM, and to determine the disparities between the primary tumor before brachytherapy and enucleated samples after brachytherapy. WES of 19 samples (13 primary tumors, 5 enucleation samples after brachytherapy, and 1 liver metastasis) from 13 patients diagnosed with UM and treated between 2007 and 2019 at the Yonsei University Health System (YUHS) were analyzed using bioinformatics pipelines. We identified significantly altered genes in Asian UM and changes in mutational profiles before and after brachytherapy using various algorithms. GNAQ, BAP1, GNA11, SF3B1 and CYSLTR2 were significantly mutated in Asian UM, which is similar that reported frequently in previous Western-based UM studies. There were also similar copy number alterations (M3, 1p loss, 6p gain, 8q gain) in both groups. In paired comparisons of the same patients, DICER1 and LRP1B were distinctly mutated only in tumor samples obtained after brachytherapy using rare-variant association tests (*P* = 0.01, 0.01, respectively). The mutational profiles of Asian UM were generally similar to the data from previous Western-based studies. DICER1 and LRP1B were newly mutated genes with statistical significance in the regrowth samples after brachytherapy compared to the primary tumors, which may be related to resistance to brachytherapy.

## Introduction

Uveal melanoma (UM) is the most common primary intraocular malignancy in adults. The incidence of UM varies widely between races. In Western populations, the annual incidence is 5 to 10 per million population per year^1,2^, whereas in Asia, it is reportedly much lower at 0.4 to 0.6^3,4^. Various radiotherapeutic and local therapeutic options have been applied to UM. The Collaborative Ocular Melanoma Study (COMS) trial showed that UM-related mortality rates were not significantly different between enucleation and plaque brachytherapy in the patients with medium-sized UM^5,6^. The results justified the use of plaque radiotherapy rather than enucleation for most medium-sized UMs. Since then, this treatment has been expanded to apply to small as well as large UMs. Despite these treatment efforts, in over one-quarter to one-third of patients, metastasis develops within 10 years, usually involving the liver, and death typically occurs 1–3 years after treatment^7,8^. Until now, the mutation profiles of Asia UM were little known; for the first time, we performed whole exome sequencing (WES) in Asian UM, and compared the results with the Cancer Genome Atlas (TCGA)^9^ data, which is the largest cohort composed mainly of Western patients. In addition, we compared the mutation profiles by WES before and after brachytherapy in eyes enucleated due to post-brachytherapy tumor regrowth. Schematic diagram of workflow is written in Supplementary Fig. 1.

## Results

### Patient characteristics

This study included 13 UM patients that have been diagnosed and treated at the Yonsei university health system (YUHS) from August 2007 to December 2019. The study was approved by the Institutional Review Board (IRB) at YUHS. and written informed consent was obtained All study protocols adhered to the tenets of the Declaration of Helsinki.

All patients were treated with brachytherapy after local resection. Enucleation was offered as the principal treatment if the patient had a large tumor (height > 10.0 mm), however, the patients who strongly refused primary enucleation received the brachytherapy. Local resection was performed for tumor debulking or diagnostic confirmation by endoresection for choroidal tumors and exoresection for iris or ciliary body tumors. Afterwards, brachytherapy with 106 Ru plaques (Eckert & Ziegler BEBIG, Berlin, Germany) was performed, with target tumor apex radiation doses ranging 85–100 Gy. When local recurrence (tumor regrowth) was noted during follow up, enucleation was performed.

Demographical and clinical data are summarized in Table 1. The analyzed samples were composed of triple types of samples (primary, enucleation after brachytherapy, and liver metastasis). Overall, 13 samples were primary tumors and 5 were paired enucleation samples after brachytherapy. Triple -paired samples were obtained from one patient.

**Table 1:** Summary of demographical and clinical data in enrolled uveal melanoma patients.

Variable	Enrolled Patients (n = 13)
**Age at diagnosis, years**	
Mean	50.2
Median (range)	54.0 (32–73)
**Sex, No. (%)**	
Female	3 (23.1)
Male	10 (76.9)
**Tumor location, No (%)**	
Choroid	8 (61.5)
Ciliary body	3 (23.1)
Iris	2 (15.4)
**Tumor Height, mm**	
Mean	10.5
Median (range)	10.8 (7.4–12.7)
NA	1
**Brachytherapy, No (%)**	
Yes	11 (84.6)
No	2 (15.4)
**Enucleation after Brachytherapy, No (%)**	
Yes	8 (61.5)
No	5 (38.5)
**Metastasis, No (%)**	
Yes	7 (46.2) (Choroid: 5; Ciliary body: 2; Iris: 0)
No	6 (53.8)
**Follow-up, months**	
Mean	79.5
Median (range)	79.9 (19.5–140.6)

### Somatic variant detection

Genome Analysis Tool kit (GATK)^10^ 4.1.0.0. Mutect2^11^ without matched normal pipeline was used to call somatic variant. The average target region sequencing depth and the average of ontarget rate of preprocessed bam were 87.21x (SD 11.28) and 96% (SD 0.5%), respectively. The sequencing coverage and quality statistics are provided in Supplementary data.

Since we have called variant of FFPE samples without matched blood samples, the optimized filtration pipeline for tumor only sequencing^12^ was applied applied on somatic variant candidates from MuTect2. After filtration to distinguish somatic alterations from germline and sequencing artifacts, the average number of somatic alterations of primary samples (n = 13) was 21.46 (SD 12.38). This was not statistically different to the number of somatic mutations in TCGA UM (Wilcoxon Rank Sum test, *P* = 0.203, Supplementary Fig. 2).

### Significant mutated gene analysis

**Figure 1:**
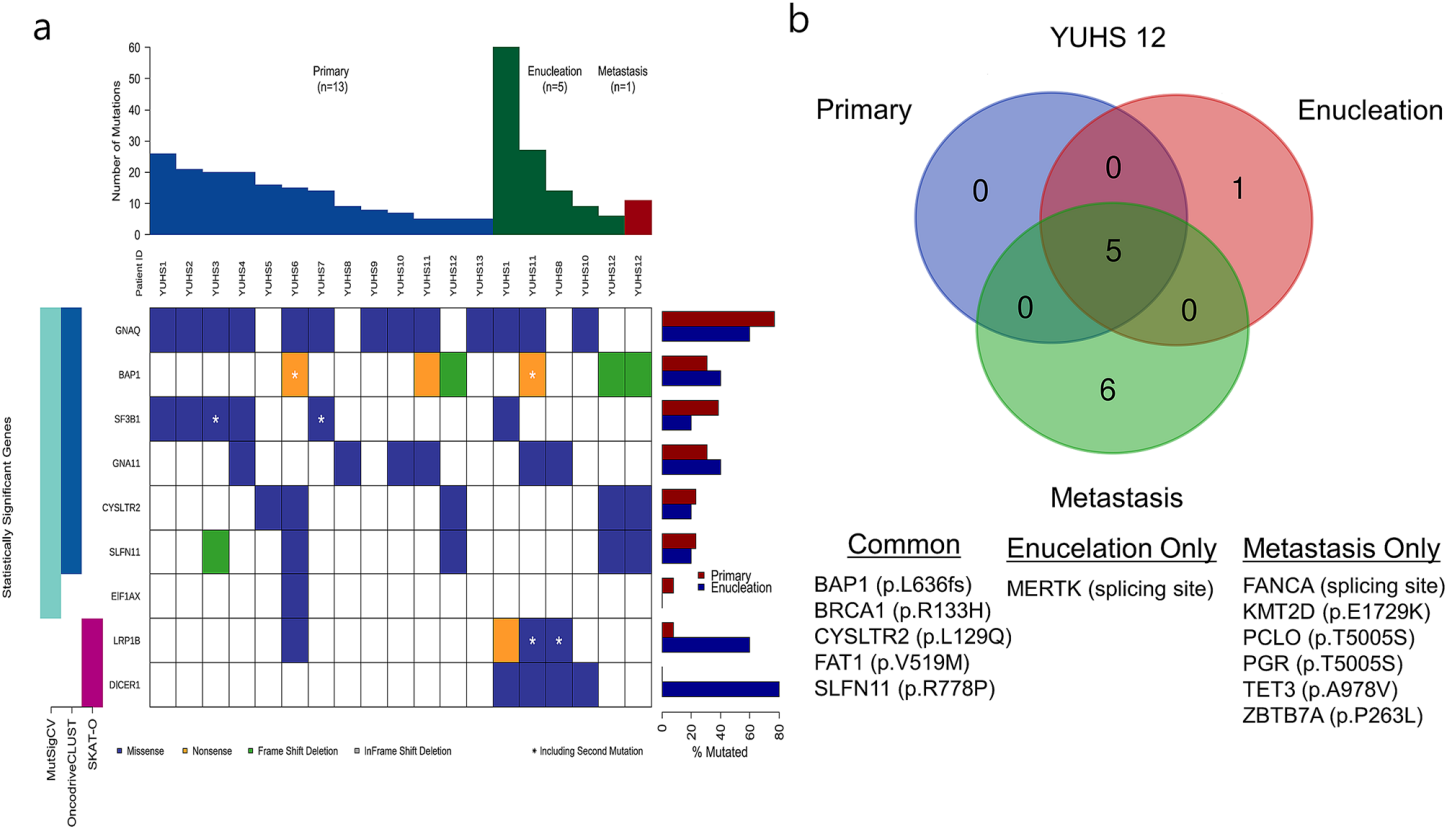
(**a**) Summary of Enrolled Uveal Melanoma Exome Analysis. The nonsynonymous somatic altered genes are color coded in waterfall plot. Left color coded bar indicates genes that significant in each tests such as MutSigCV, OncodriveCLUST and SKAT-O. (**b**) Comparison of somatic alterations in primary, enucleation and metastasis from YUHS 12 patient.

In MutSigCV, a total of 10 genes were found to be significant in the YUHS data (Fig. 1A, Supplementary Fig. 3). GNAQ, BAP1, GNA11, SF3B1, EIF1AX, PTPRD and CYSLTR2 were similarly found to be significant genes in the TCGA data using the same algorithm, whereas the other 3 genes (SLFN11, KTN1 and FANCL) were not. Using the OncodriveCLUST algorithm, 6 genes (GNA11, BAP1, GNAQ, SF3B1, CYSLTR2 and SLFN11) were noted to be significant in the YUHS data (Fig. 1A, Supplementary Fig. 4); and except for BAP1, CYSLTR2 and SLFN11, these were also significant in the TCGA data. Altogether, GNAQ, BAP1, SF3B1, GNA11, and CYSLTR2 were significant in both algorithms, and have been reported as frequently mutated genes in a previous UM study^13^. We could find common recurrent alterations (p.Q209P/L of GNAQ, p.Q209L of GNA11, p.L129Q of CYSLTR2 and p.R625H/C of SF3B1) in both datasets (Supplementary Figs. 5–10).

### Copy number alterations

In previous UM studies^13,14^, recurrent copy number alterations (CNAs) of chromosome 1p, 3, 6p, and 8q have been reported. Those CNAs were also observed in YUHS UMs (Supplementary Fig. 11). There were 3 YUHS samples (23%) and 22 TCGA samples (27.5%) with chromosome 8q gain (Supplementary Fig. 12). The loss of chromosome 1p were found in 2 YUHS UMs (15.4%) and 21 (26.2%) TCGA UMs. The proportion of the two CNAs in YUHS UMs was similar with TCGA UM (Fisher’s exact test; 8q gain, *P* = 1.00; 1p loss, *P* = 0.51). On the other hand, there were 6 YUHS UMs (46.1%) and 17 TCGA UMs (21%) with chromosome 6p gain (*P* = 0.02). While two samples (15.4%) with monosomy chromosome 3 (M3) were found in YUHS UM samples, 42 samples (52.5%) had M3 in the TCGA UMs cohort (*P* = 0.03).

### Molecular changes between before and after brachytherapy

In paired comparisons using the SKAT-O test, DICER1 and LRP1B were newly mutated genes with statistical significance in enucleation samples when compared to the primary tumor of the same patients (*P* = 0.01 and 0.01, respectively; Fig. 1A). The 4 types of somatic alterations of DICER1 and 5 somatic alterations of LRP1B were exclusively present in the enucleation samples (Table 2).

**Table 2:** Altered variants of SKAT-O Significant Genes (DICER1, LRP1B) in paired samples.

Gene	*P* Value	Chromosome	Position	REF	Alt	Amino acid	SIFT	Polyphen2	CADD
DICER1	0.010	14	95,560,248	C	T	p.E1781K	0.1	0.818	23
		14	95,562,263	C	T	p.G1665E	0	0.916	29.4
		14	95,570,303	C	T	p.E1144K	0.03	0.19	20.4
		14	95,577,763	C	T	p.G716E	0.002	0.832	34
LRP1B	0.021	2	141,128,318	C	T	p.D3657N	0.02	0.832	26.6
		2	141,242,964	C	A	p.V3125F	0.01	0.312	33
		2	141,763,012	G	A	p.R799X	1	NA	38
		2	141,986,887	C	T	p.D239N	0	0.08	26.9
		2	142,012,117	A	T	p.I146K	0.01	0.184	23.6

*Ref* reference base, *Alt* alternative base, *SIFT* sorting intolerant from tolerant, *CADD* combined annotation dependent depletion.

In a triple comparison of one patient (YUHS 12), comprising of the primary tumor, an enucleated sample after brachytherapy, and a liver metastasis sample, when the latter samples were compared to the primary tumor, one gene (METRK) mutations in the enucleation sample and six gene (FANCA, KMT2D, PCLO, PGR, TET3, and ZBTB7A) mutations in the metastasis were found to be newly occurred, and were each exclusive without common mutations (Fig. 1B).

### Mutational signature analysis

**Figure 2:**
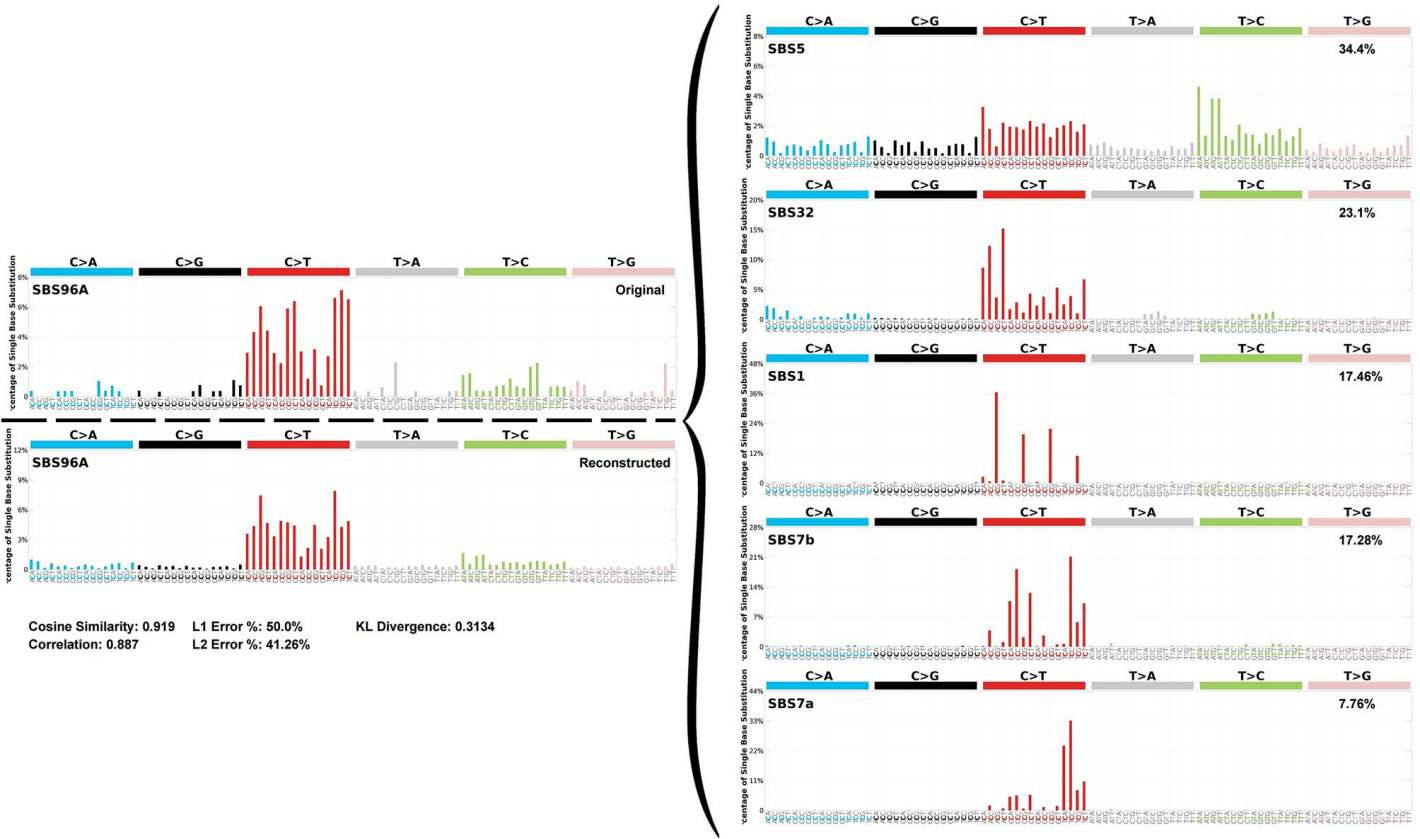
Decomposition plots of mutational signature analysis with primary samples (n = 13). Plots of Original and reconstructed data (left) and decomposed 5 mutational signature plots (right).

Mutational signature analysis with primary variants data indicated mainly 5 signatures (Cosine similarity: 0.919), which were single base substitution (SBS) 5 (34.44%), SBS32 (23.1%), SBS1 (17.46%), SBS7b (17.28%), and SBS7a (7.76%) in primary samples (Fig. 2). SBS7b and SBS7a have been found in skin cancers from sun exposed areas and thus likely to be due to exposure to ultraviolet (UV) light. However, none of the signatures from TCGA UM was related to exposure to UV (Supplementary Fig. 13). Additionally, SBS5 was also found in TCGA UM signatures and SBS1 has been known to be related to various malignancies and aging. Therefore, we found both known signatures (SBS1, SBS5) from previous Western-based UM studies. Similar to a previous study showing ultraviolet radiation signatures were observed in iris tumors^14^, SBS7b and SBS7a were predominantly (≥ 50%) observed in YUHS iris melanoma samples (YUHS7, YUHS9) (Supplementary Fig. 14).

## Discussion

We analyzed Asian UM and confirmed that significantly mutated genes were similar to those of TCGA, the largest western cohort.

In primary sample analysis, we found similar mutational profiles with western data. The 5 genes (GNAQ, BAP1, SF3B1, GNA11 and CYSTLR2) that have been reported as recurrent mutated or driver genes in other UM study were significant in two algorithms (MutsigCV and OncodriveCLUST). In addition, SLFN11 that never have been reported as UM related gene was also significant in both of them. This could be novel UM related cancer gene. The previously reported recurrent CNAs of UM were similarly observed in Asian UMs. Although the proportion of some CNAs (6p gain, M3) was different between the two cohorts, it is difficult to determine whether the results are from a difference between Asian and Western populations due to the small sample size of our cohort.

In the cases where the tumor regrows post-brachytherapy, enucleation should be considered. We performed paired comparison of WES data before and after brachytherapy and confirmed that two novel genes, DICER1 and LRP1B, were significantly mutated in the regrowth samples compared to the primary tumors. These two genes have already been associated with malignancy in non-ocular tumors^15^. It is not clear whether these newly developed mutations are the result of cancer evolution or the effect of brachytherapy; however, they could be related to resistance to brachytherapy.

In a triple comparison of one patient (Fig. 1B), the enucleated regrowth sample after brachytherapy and liver metastasis sample did not share any newly developed mutations. This may indicate that the liver metastasis event occurred earlier and independently of the tumor regrowth after brachytherapy.

## Conclusion

This report, albeit a small sample size, is the first WES analysis in Asian UM, and through paired comparisons, novel and insightful results can be drawn.

## Methods

### Sample preparation and data generation

DNA was extracted from 19 formalin fixed paraffin embedded (FFPE) tissue from 13 UM patients that have been diagnosed and treated at the Yonsei university health system (YUHS) from August 2007 to December 2019 and captured using SureSelectHuman All Exon V6 for whole exome sequencing. After library QC of DNA, the libraries were sequenced on the Illumina HiSeq X ten platform. This study was approved by the Institutional Review Board (IRB) at YUHS and written informed consent was obtained. All study protocols adhered to the tenets of the Declaration of Helsinki.

### Sequencing data processing and somatic variant analysis

The paired end reads of FASTA files from sequencing were aligned using BWA-mem^16^ on human genome (hg) 19. After duplicated reads of the aligned bam files were marked and removed using Picard, the base quality of reads in bam files was recalibrated using Genome Analysis Tool kit (GATK)^10^ 4.1.0.0. Mutect2^11^ without matched normal mode was used to call somatic variant candidates with gNomad^17^ as germline resources.

### Variant filtration for only tumor sequencing and Annotation

To remove sequencing artifact, the variants with "bad_haplotype", "chimeric_original_alignment","base_quality","duplicate_evidence","fragment_length","low_avg_alt_quality","mapping_quality","multiallelic","n_ratio","read_orientation_artifact", "read_position", "str_contraction","strand_artifact" and "strict_strand_bias" were filtered out. We selected genes that have been reported as cancer genes in both OncoKB^18^, cBioportal^19^, for uveal melanoma. We also added MAPKAPK5 which was not listed in OncoKB but it has been reported to be a frequently mutated gene in uveal melanoma data from TCGA. If the population allele frequency of the variants is more than 1% in any subpopulation of 1000 genomes project^20^, ExAC^21^, KOVA^22^, gNomad and Korean Genome Project^23^ data, the variants were excluded as germline mutations. The second exclusion criteria is that the variant were present in the Korean 1,000 depression exome data^24^, which is a panel of normal. It removes not only germline mutation but also potential platform specific artifacts. Then we reviewed the all variants using Integrative Genomics Viewer^25^. If there is a missed variant that is found in another tissue from a same patient, we used blastn^26^ to align all sequence on neighboring region(± 50 bp) of the variant. We consider the variant exist if more than each 2 paired aligned reads that encoding same nucleotide of variants. The variants which passed the above criteria were selected as analysis ready somatic variants. All passed variants were annotated with SIFT^27^, Polylphen2^28^ and CADD^29^ score using ANNOVAR^30^.

### Significant mutated genes

The filtered non-synonymous variants were analyzed for significantly mutated genes using two algorithms, MutSigCV^31^ and OncodriveCLUST^32^. We defined the genes that have P less than 0.05 as significant in MutSicCV. on Genepattern^33^. with default parameter. OncodriveCLUST is used to identify genes with a significant bias of mutation clustering within the protein sequence. If Q value is less than 0.05 in OncodriveCLUST, the gene is significant. Maftools^34^ was used to run OncodriveCLUST. We also ran the same algorithms on uveal melanoma somatic variants data from TCGA. The somatic variant data were produced from tumor bam files from GDC data portal with same calling pipeline for YUHS.

### Identifying copy number alterations

CNVkit^35^ was used to identify copy number alterations in UMs. Because construction of a normal reference from pooled normal samples is necessary for the somatic copy number calling pipeline, 1,000 WES of normal blood from the Korean 1000 database were used. Fused Lasso (flasso) algorithm was used as a segmentation method. The thresholds of log2 transformed relative ratio to reference ploidy for copy number 0,1,2,3 were -1.1, -0.4, 0.3, 0.7, respectively. The same pipeline was also utilized on TCGA tumor-normal paired data.

### Rare variant association test

Since single variant association test has little power for testing of rare variants in relatively small samples, an alternative approach is needed. Sequence kernel association optimal test (SKAT-O)^36^ is an optimal unified approach for rare variant association testing in case control sequencing studies. The test was applied on non-synonymous variants of genes between primary and matched enucleation samples. R library SKAT was used to run the statistical test.

### Mutational signature analysis

The 96 different contexts of single base substitutions from filtered variants were analyzed for generating mutational signatures. The signatures are identified as causes of mutational process, due to their unique mutational pattern and specific activity on the genome. The python packages, SigProfilerMatrixGenerator and SigProfilerExtractor, were used for this analysis. The iteration time was set 100 times to be performed to extract each signature.

## Supplementary Information

Supplementary Information 1.

Supplementary Information 2.

## Data Availability

All data generated or analyzed during this study are included in this article. Raw sequence data have been deposited in Sequence Read Archive under PRJNA701837https://dataview.ncbi.nlm.nih.gov/object/PRJNA701837?reviewer=u0rm0asqvhrug0vv24usm3eijf.
