# Supplementary material for: Paired comparisons of mutational profiles before and after brachytherapy in asian uveal melanoma patients
Source: Sci Rep. 2021 Sep 20;11:18594. doi: 10.1038/s41598-021-98084-8 (PMC8452742; doi:10.1038/s41598-021-98084-8)
Supplement: Supplementary file 1 — Supplementary Information 1. [file 41598_2021_98084_MOESM1_ESM.pdf]

Paired Comparisons of Mutational Profiles Before and After Brachytherapy in Asian Uveal Melanoma Patients

Woo Seung Lee[1,†], Junwon Lee[2,*,†], Jun Jeong Choi[3], Hyun Goo Kang[2], Sung Chul Lee[4], Ju Han Kim[1,*]

[1]Division of Biomedical Informatics, Seoul National University College of Medicine, Seoul, South Korea

[2]Department of Ophthalmology, Institute of Human Barrier Research, Gangnam Severance Hospital, Yonsei University College of Medicine, Seoul, South Korea

[3]Department of Pharmacy and Yonsei Institute of Pharmaceutical Sciences, College of Pharmacy, Yonsei University, Incheon, South Korea

[4]Department of Ophthalmology, Konyang University College of Medicine, Daejeon, South Korea

†These authors share first authorship.

*These authors share corresponding authorship.

Correspondence:
Ju Han Kim, MD, PhD Seoul National University Biomedical Informatics (SNUBI), Division of Biomedical Informatics, Seoul National University College of Medicine, Seoul, South Korea 03080. Tel.: (+82) 2-740-8320; Fax: (+82) 2-747-8928;
E-mail Address: juhan@snu.ac.kr

Junwon Lee, MD, PhD Department of Ophthalmology, Gangnam Severance Hospital, Institute of Vision Research, Yonsei University College of Medicine, Seoul, South Korea 06273 Tel.: (+82) 2-2019-3446; Fax: (+82) 2-3463-1049;
E-mail: bravewon@yuhs.ac

Supplementary Contents

Supplementary Figure 1. Schematic diagram of workflow

Supplementary Figure 2. Density plot of somatic alteration counts in primary tumor samples from Korean (n=13) and TCGA UM cohort (n=80) without outliers.

Supplementary Figure 3. MutSigCV Results of YUHS and TCGA data. Labeled genes are statistically significant ($P<0.05$) in the YUHS data. Red dot lines indicate $-\log_{10}(0.05)$ on each axis.

Supplementary Figure 4. OncodriveCLUST Results of YUHS(left) and TCGA(right). Dot size indicates the number of mutated samples.

Supplementary Figure 5. Lollipop plot of GNAQ in YUHS and TCGA.

Supplementary Figure 6. Lollipop plot of BAP1 in YUHS and TCGA.

Supplementary Figure 7. Lollipop plot of SF3B1 in YUHS and TCGA.

Supplementary Figure 8. Lollipop plot of GNA11 in YUHS and TCGA.

Supplementary Figure 9. Lollipop plot of CYSLTR2 in YUHS and TCGA.

Supplementary Figure 10. Lollipop plot of SLFN11 in YUHS and TCGA.

Supplementary Figure 11. Heatmap of copy number alteration status in YUHS primary UM.

Supplementary Figure 12. Heatmap of copy number alteration status in TCGA primary UM.

Supplementary Figure 13. Decomposition plots of mutational signature analysis with TCGA samples (n=80).

Supplementary Figure 14. Normalized mutational contribution of signatures per YUHS samples (n=13).

Supplementary Data. Sequencing quality statistics, somatic alterations and results of MusigCV and OncodriveCLUST of YUHS and TCGA

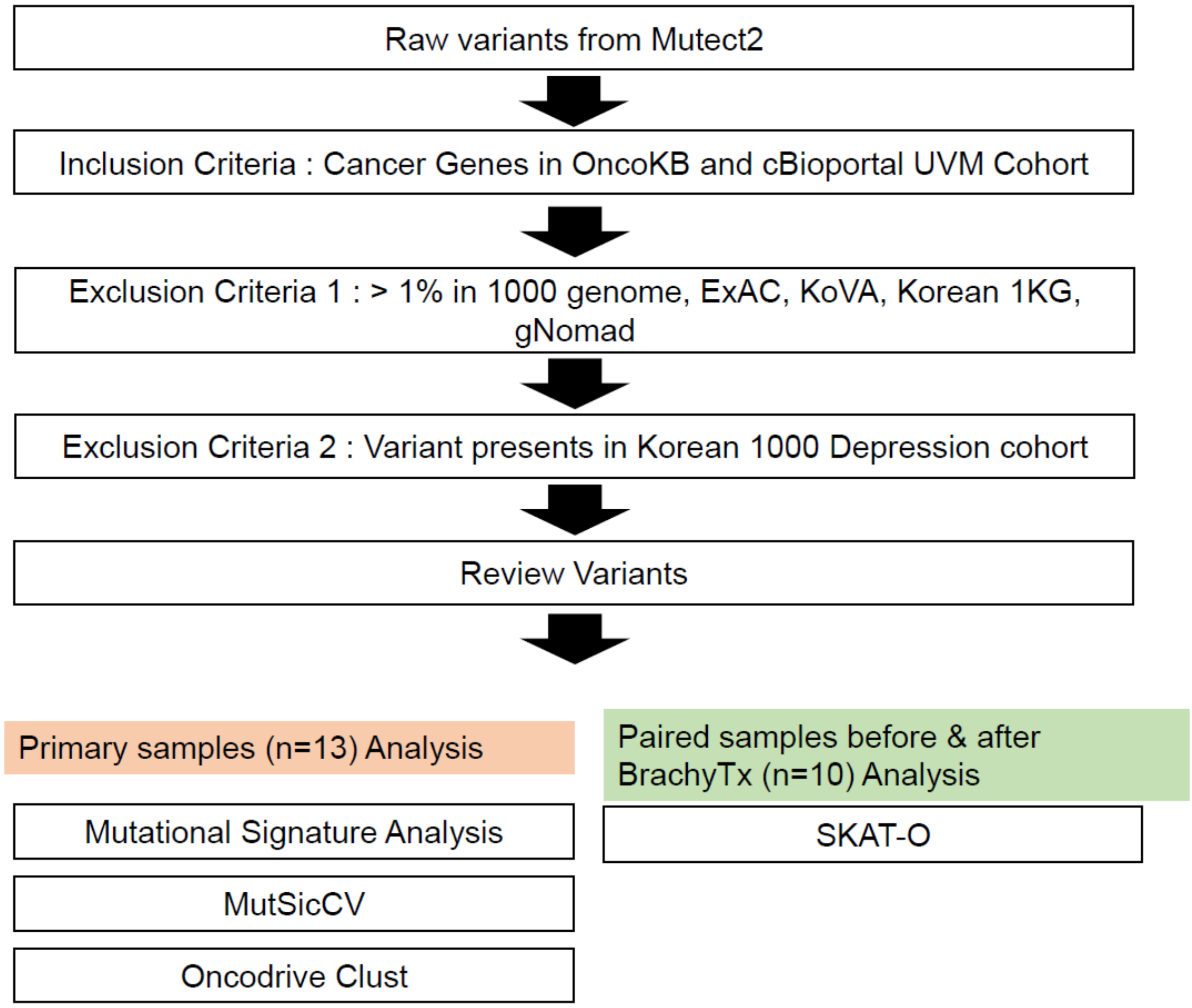

Supplementary Figure 1. Schematic diagram of workflow

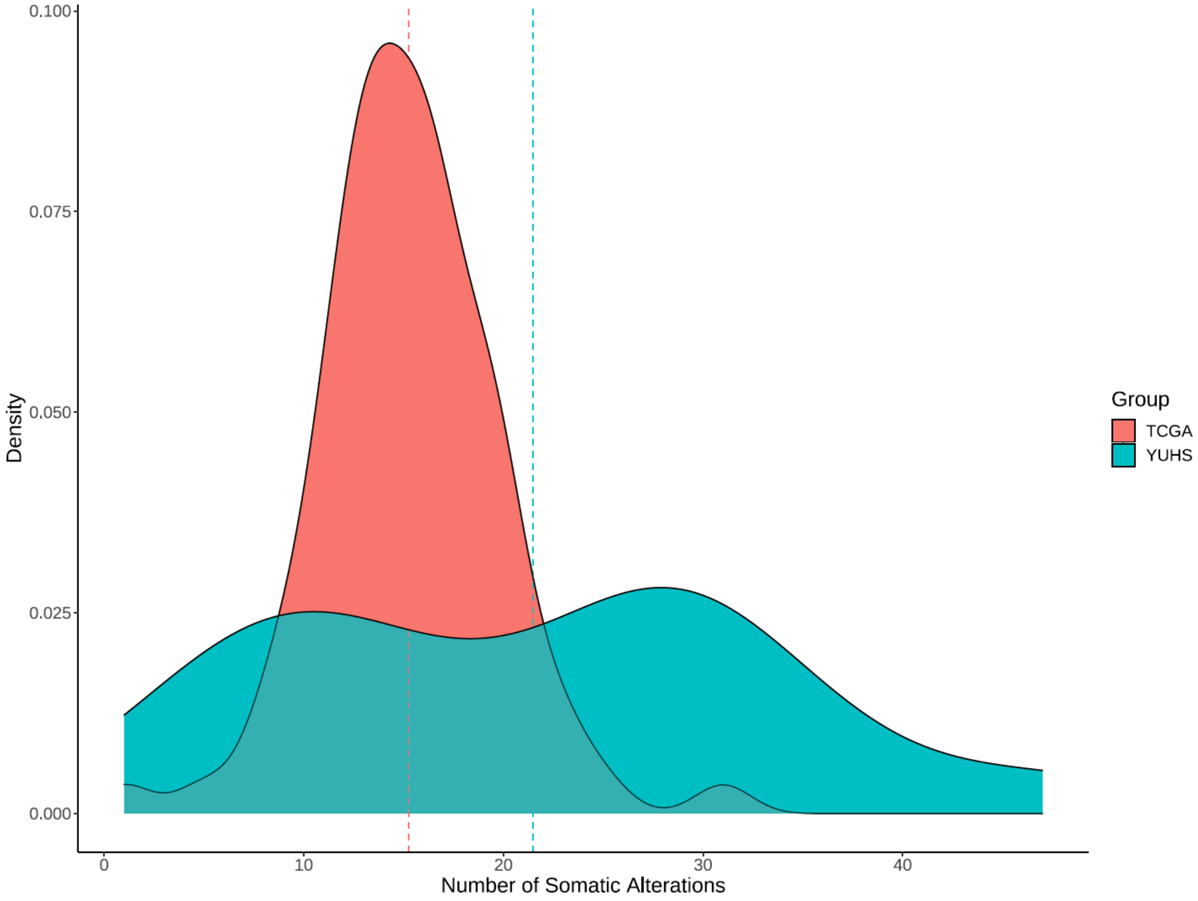

Supplementary Figure 2. Density plot of somatic alteration counts in primary tumor samples from YUHS (n=13) and TCGA UM cohort (n=80). Vertical lines indicate the average number of somatic alterations in each group.

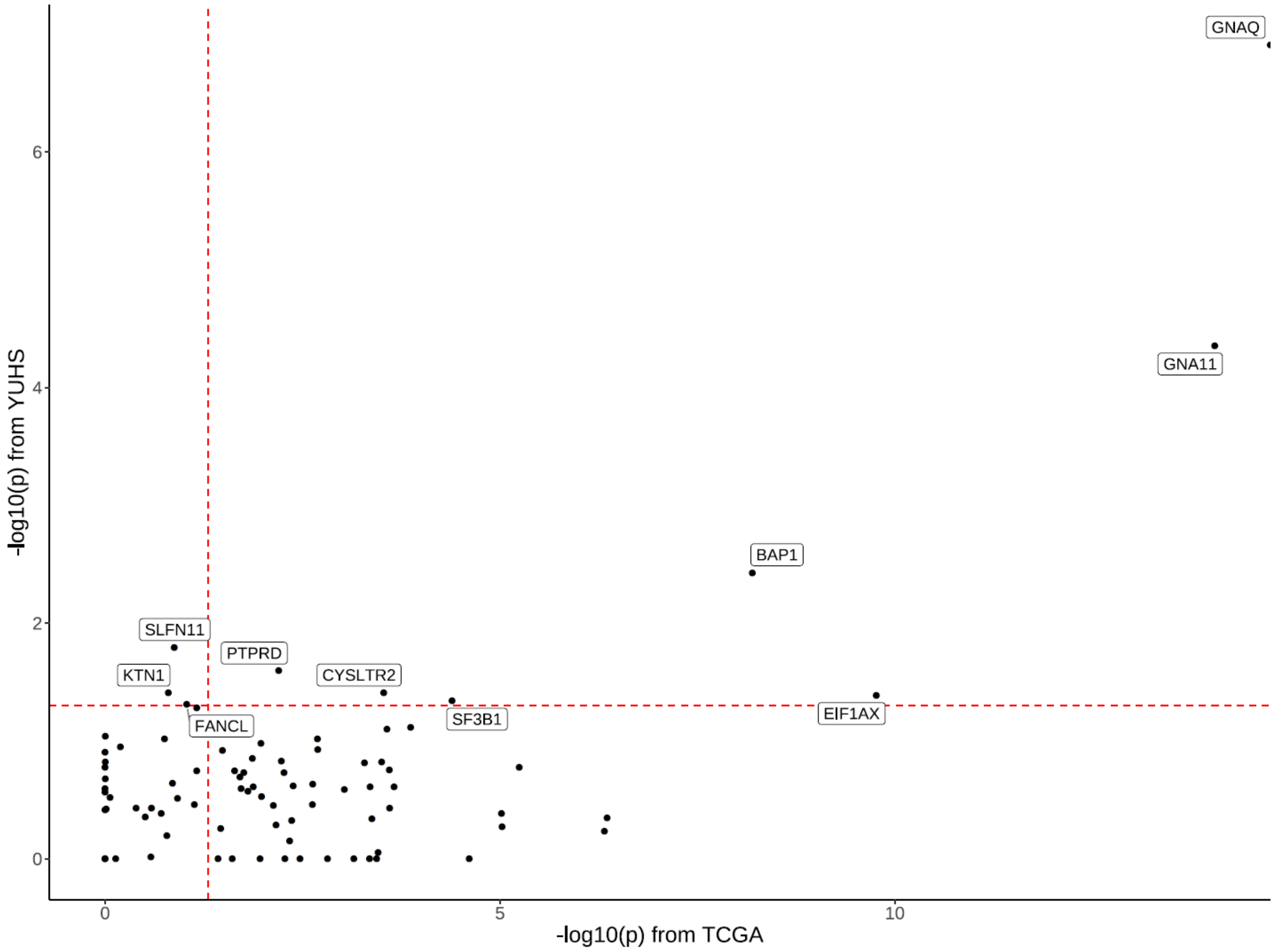

Supplementary Figure 3. MutSigCV Results of YUHS and TCGA data. Labeled genes are statistically significant (P<0.05) in the YUHS data. Red dot lines indicate -$\log_{10}(0.05)$ on each axis.

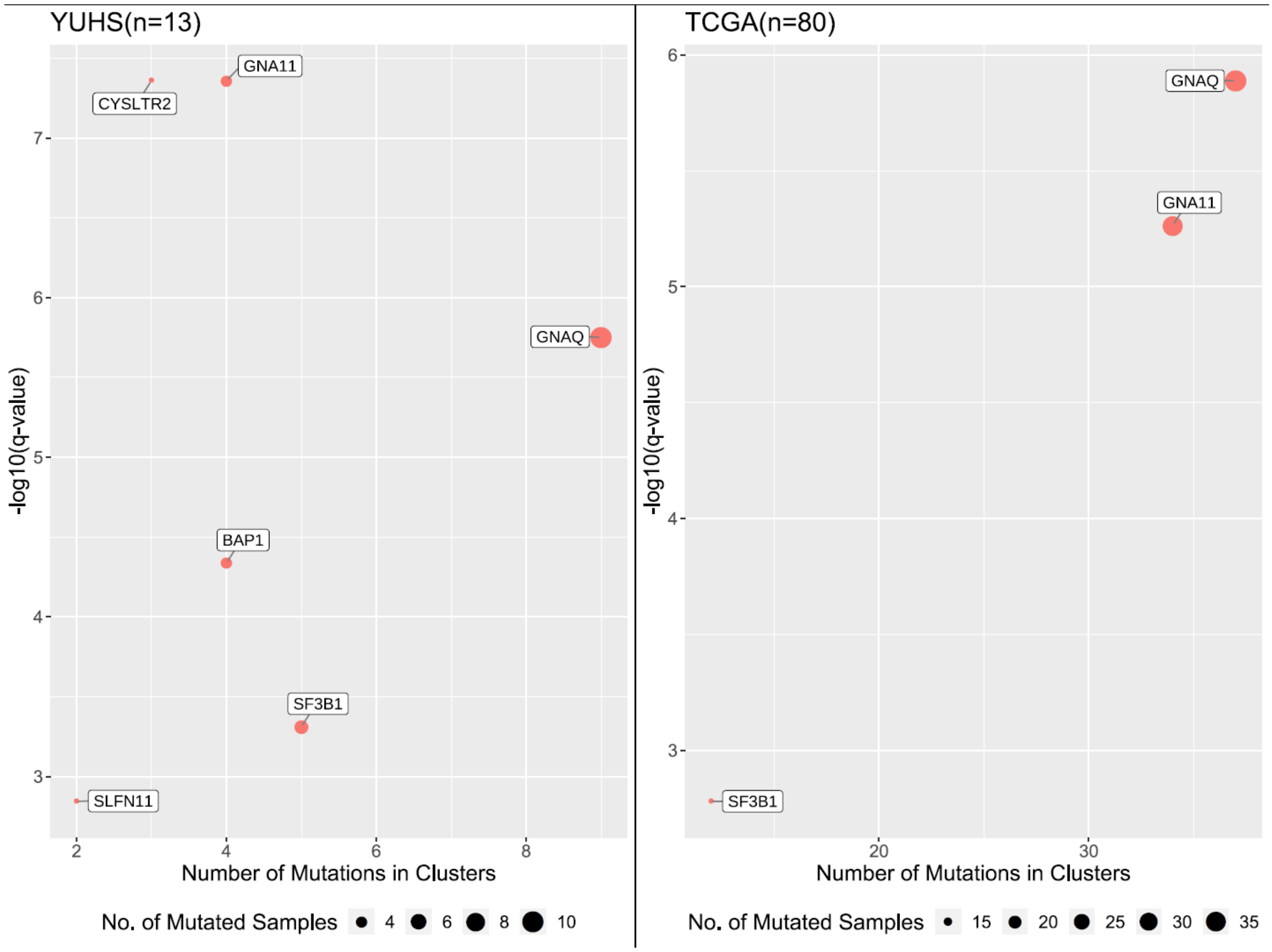

Supplementary Figure 4. Oncodrive Clust Results of YUHS(left) and TCGA(right). Dot size indicates the number of mutated samples.

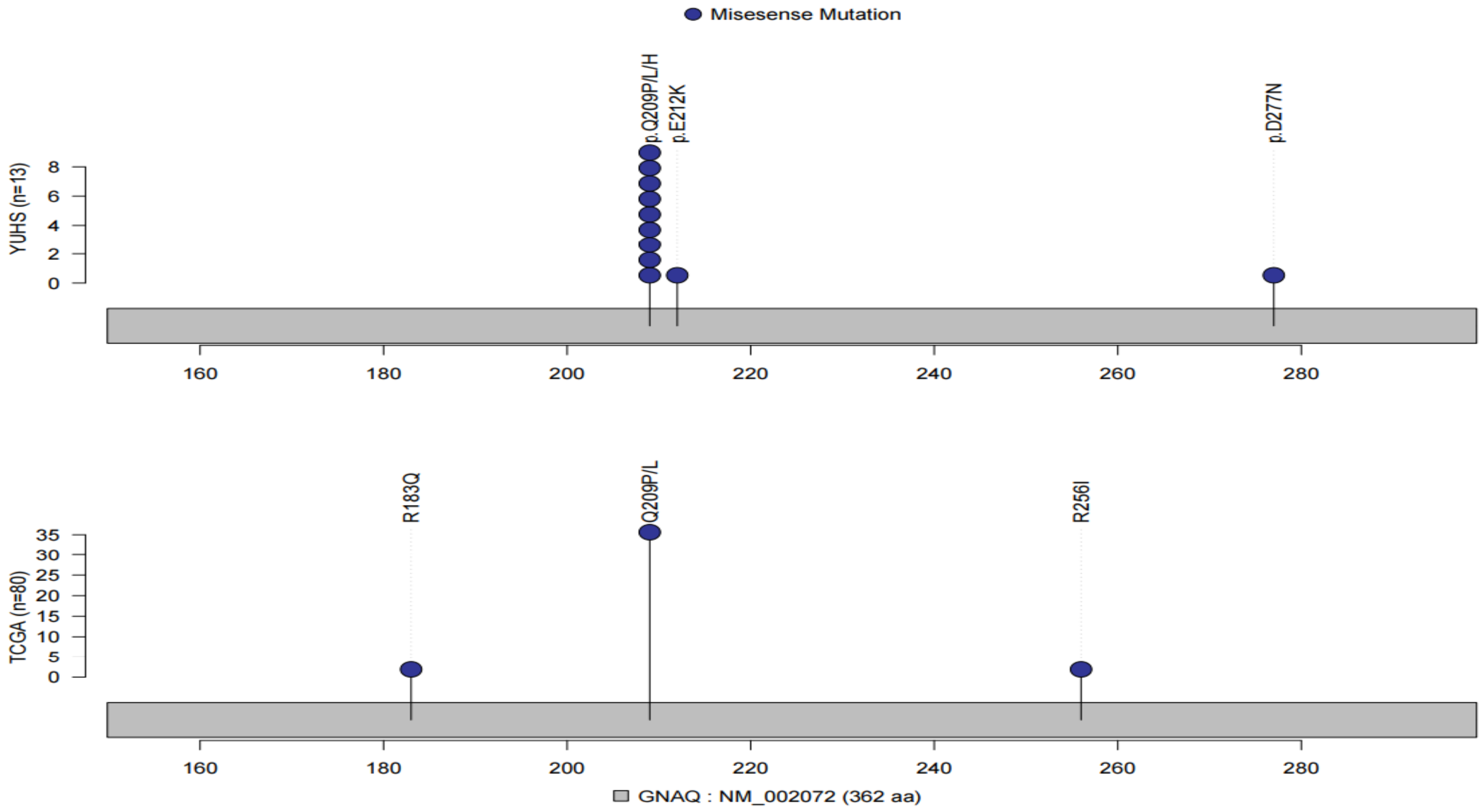

Supplementary Figure 5. Lollipop plot of GNAQ in YUHS and TCGA.

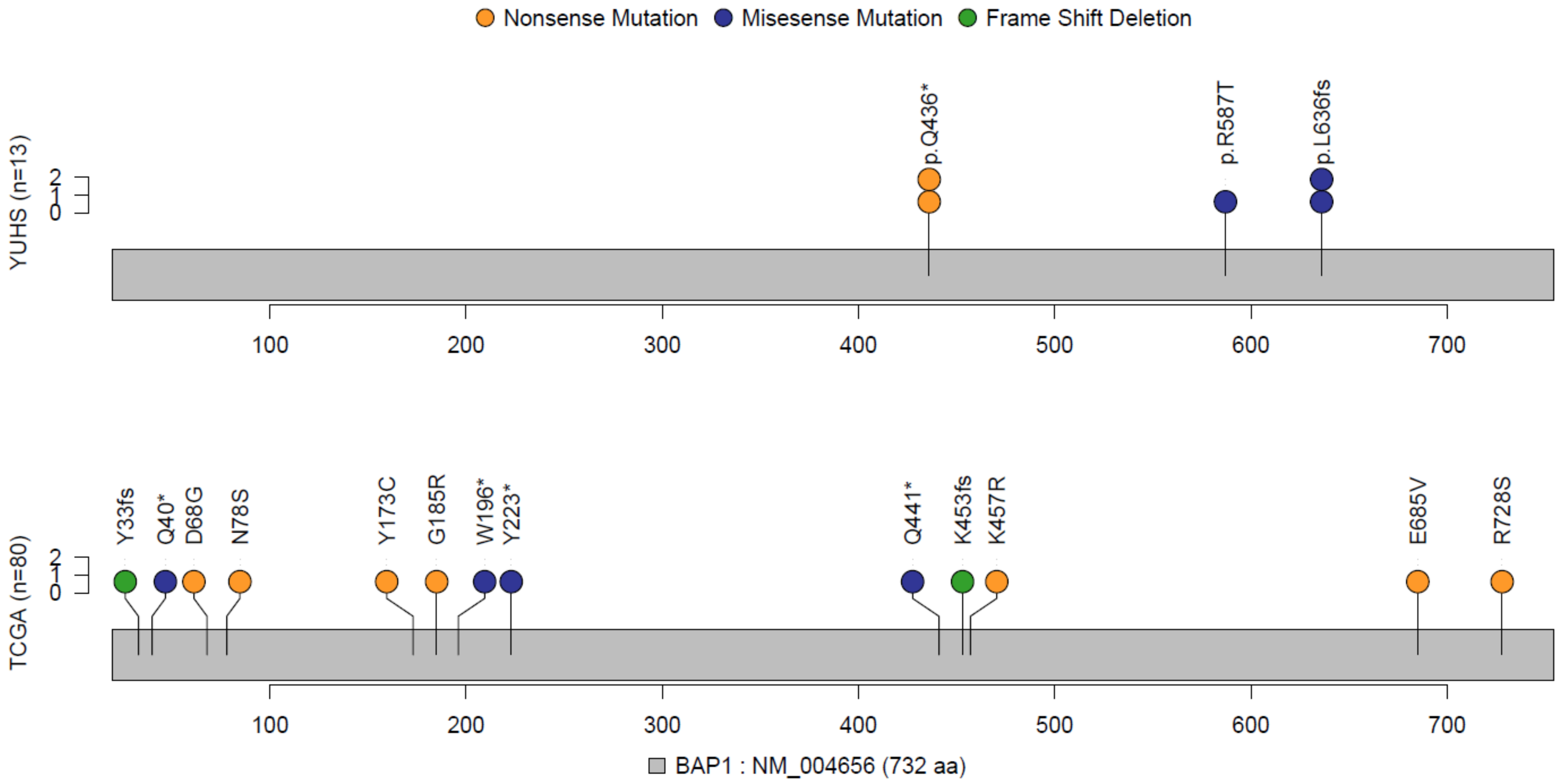

Supplementary Figure 6. Lollipop plot of BAP1 in YUHS and TCGA.

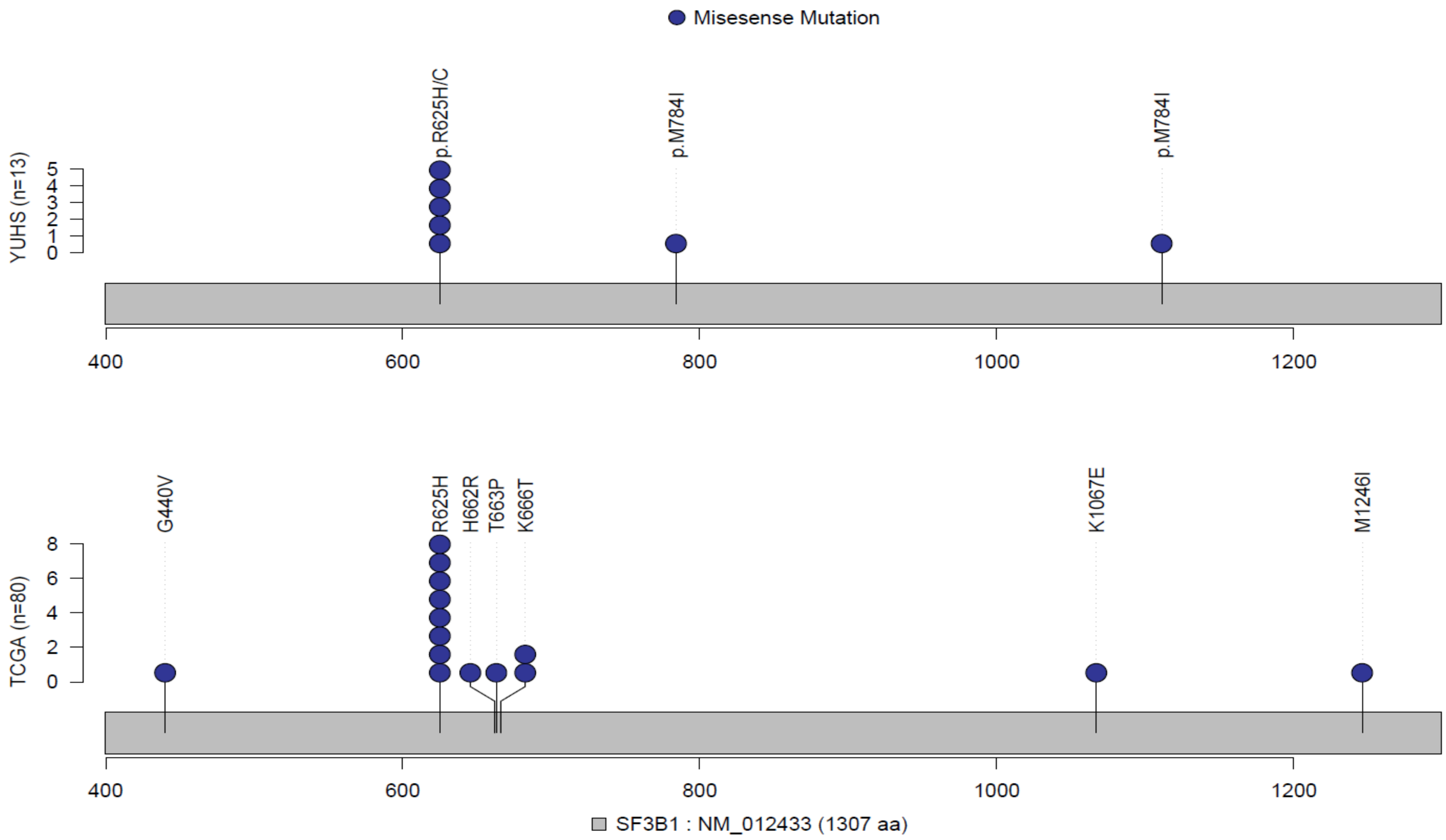

Supplementary Figure 7. Lollipop plot of SF3B1 in YUHS and TCGA.

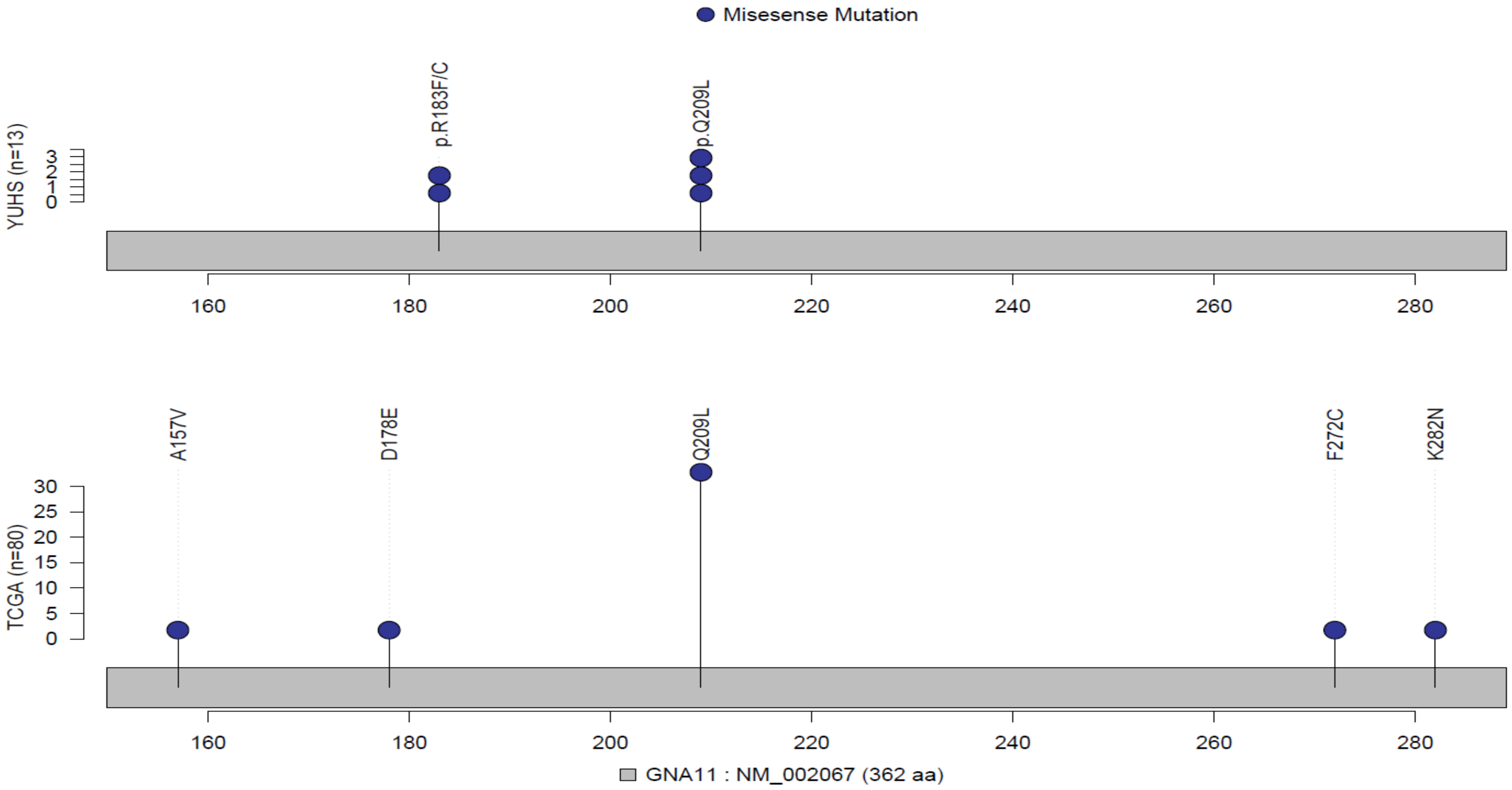

Supplementary Figure 8. Lollipop plot of GNA11 in YUHS and TCGA.

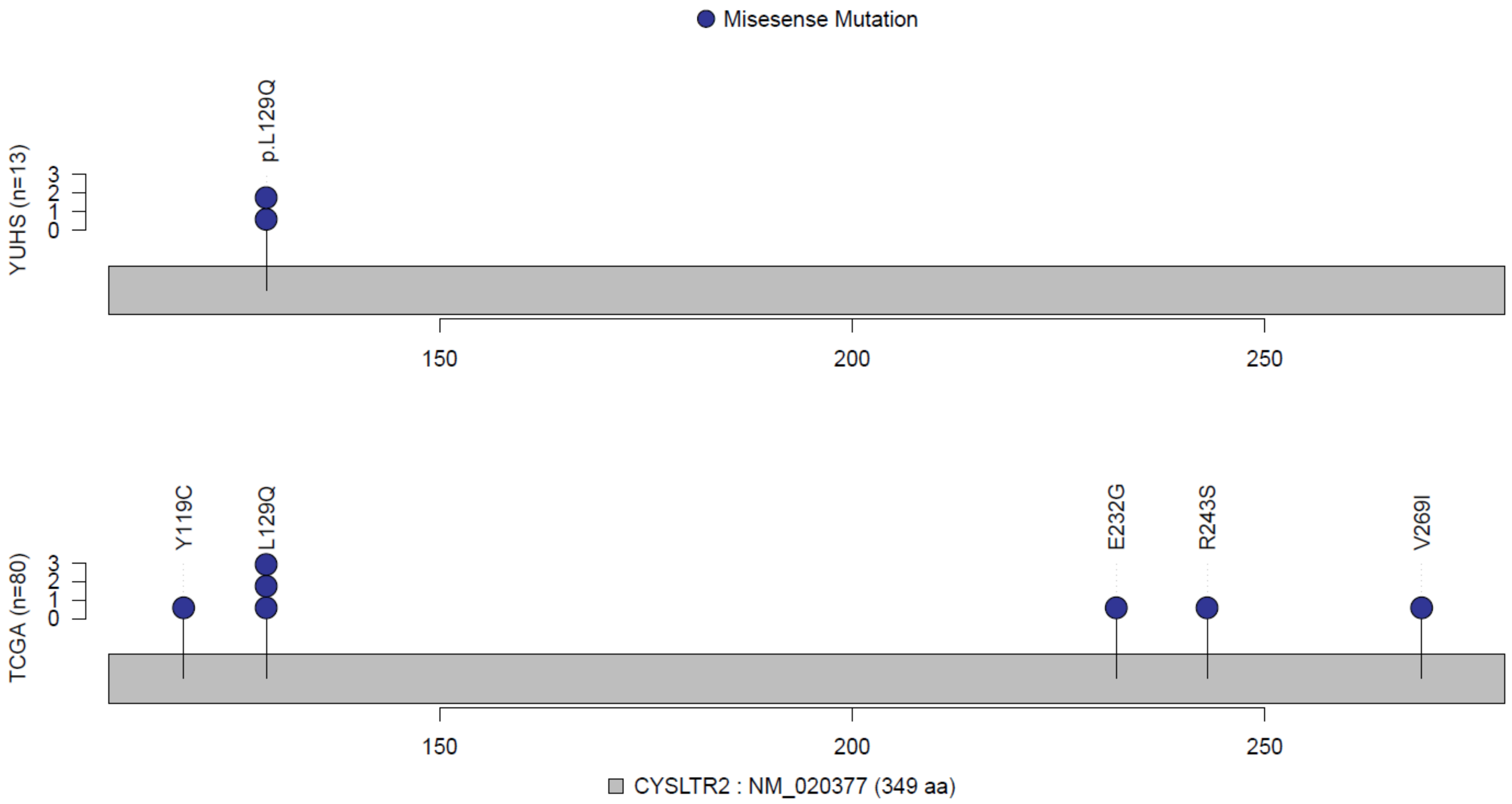

Supplementary Figure 9. Lollipop plot of CYSLTR2 in YUHS and TCGA.

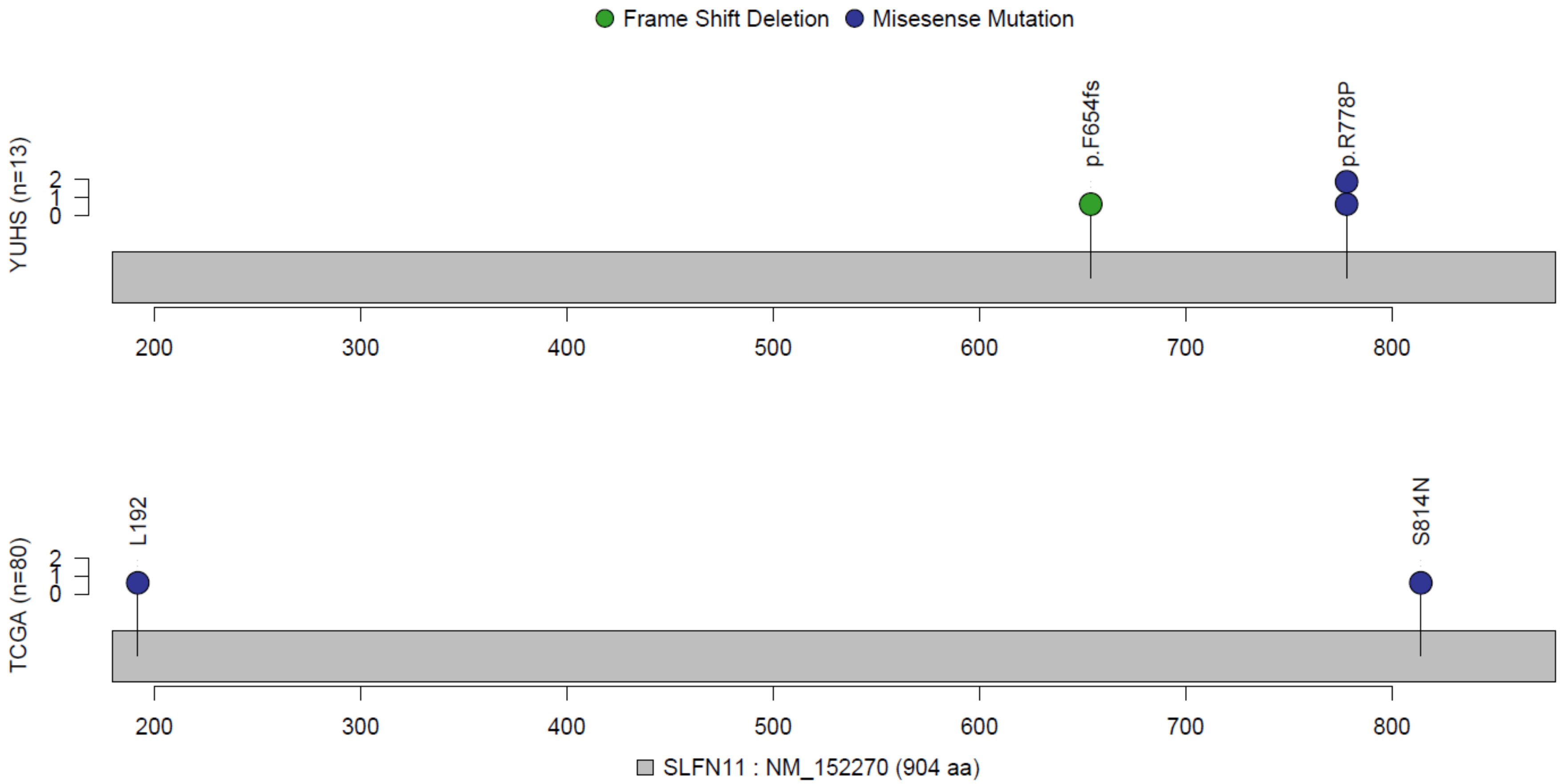

Supplementary Figure 10. Lollipop plot of SLFN11 in YUHS and TCGA.

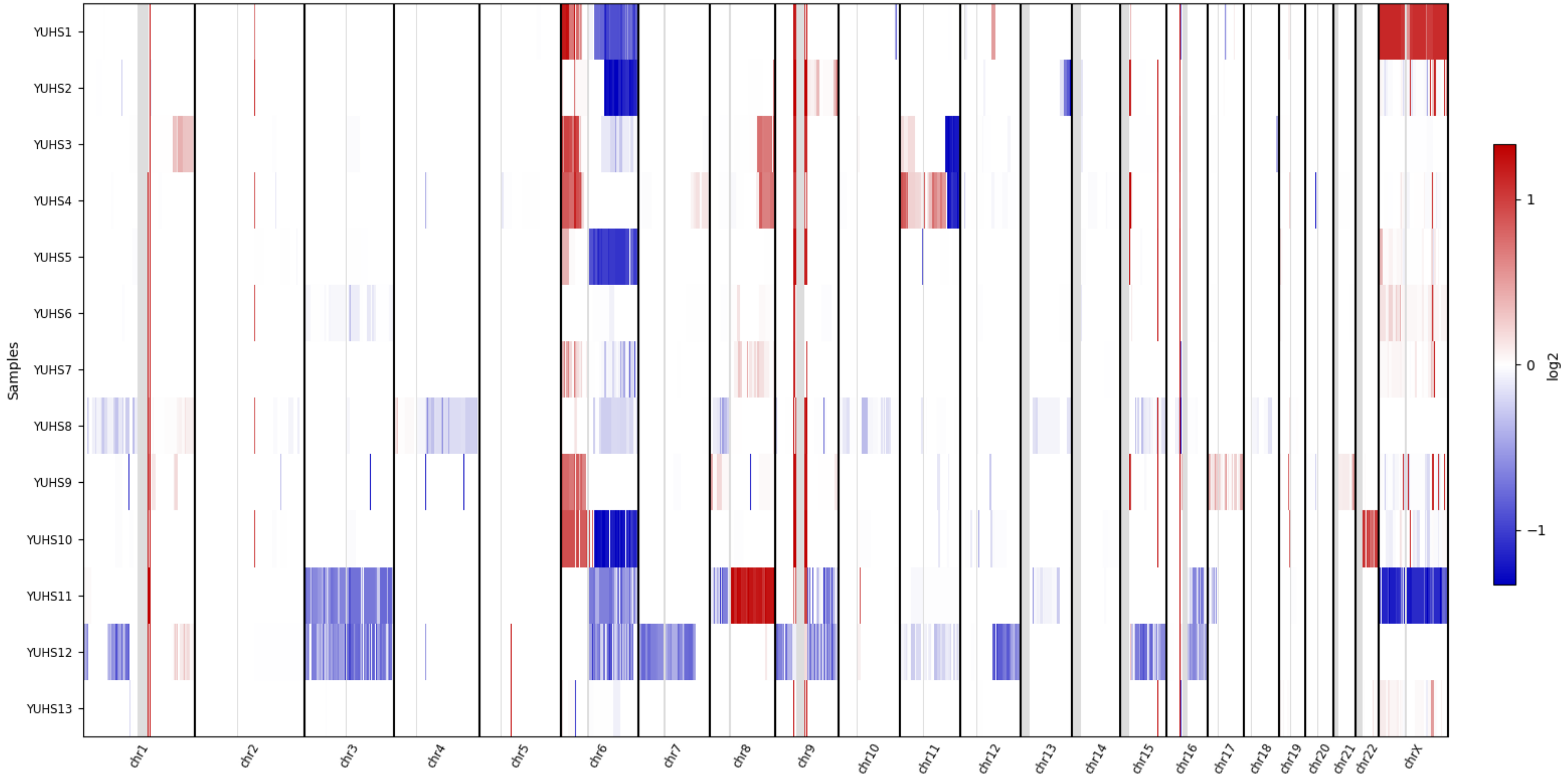

Supplementary Figure 11. Heatmap of copy number alteration status in YUHS primary UM.

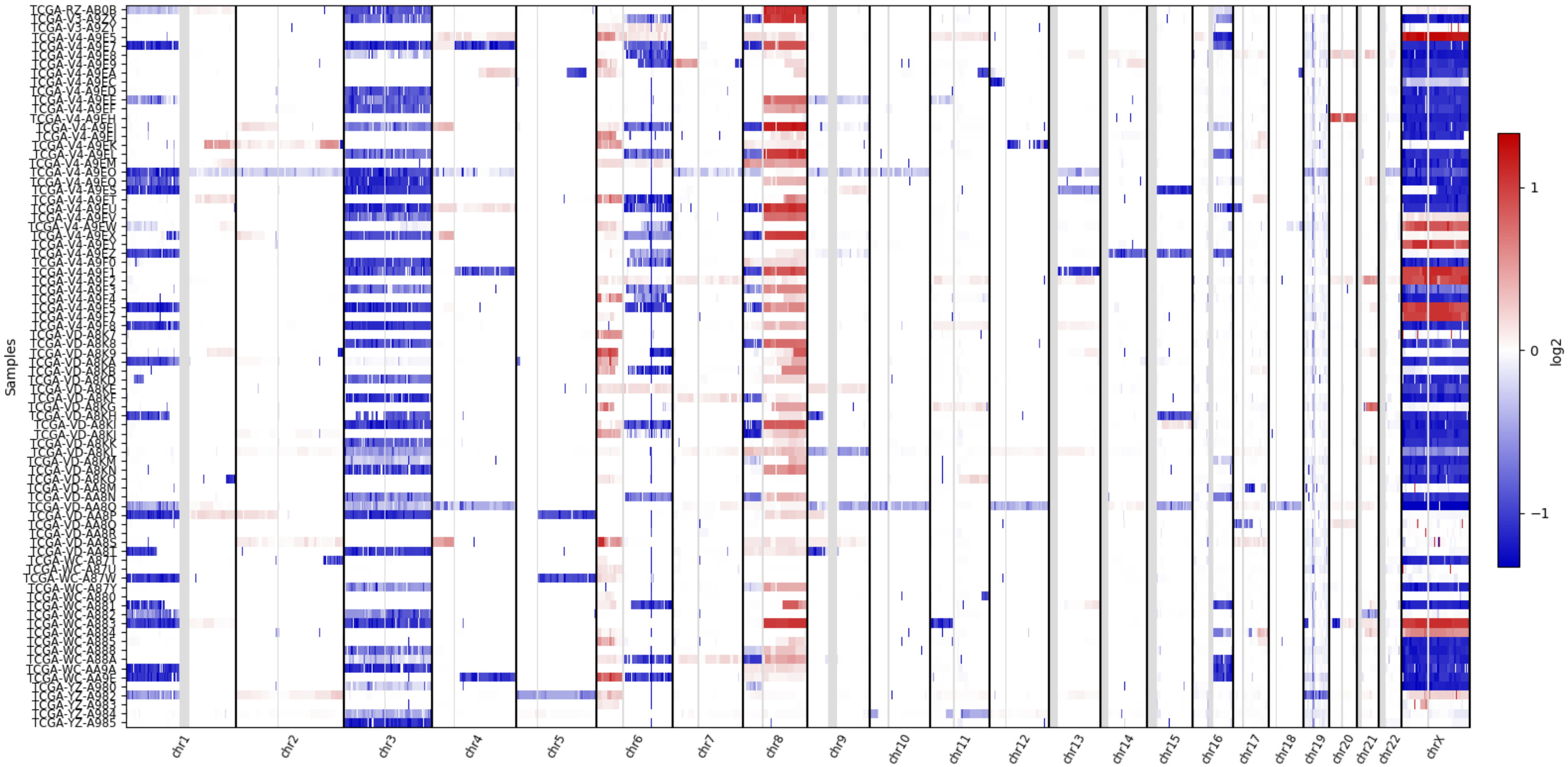

Supplementary Figure 12. Heatmap of copy number alteration status in TCGA primary UM.

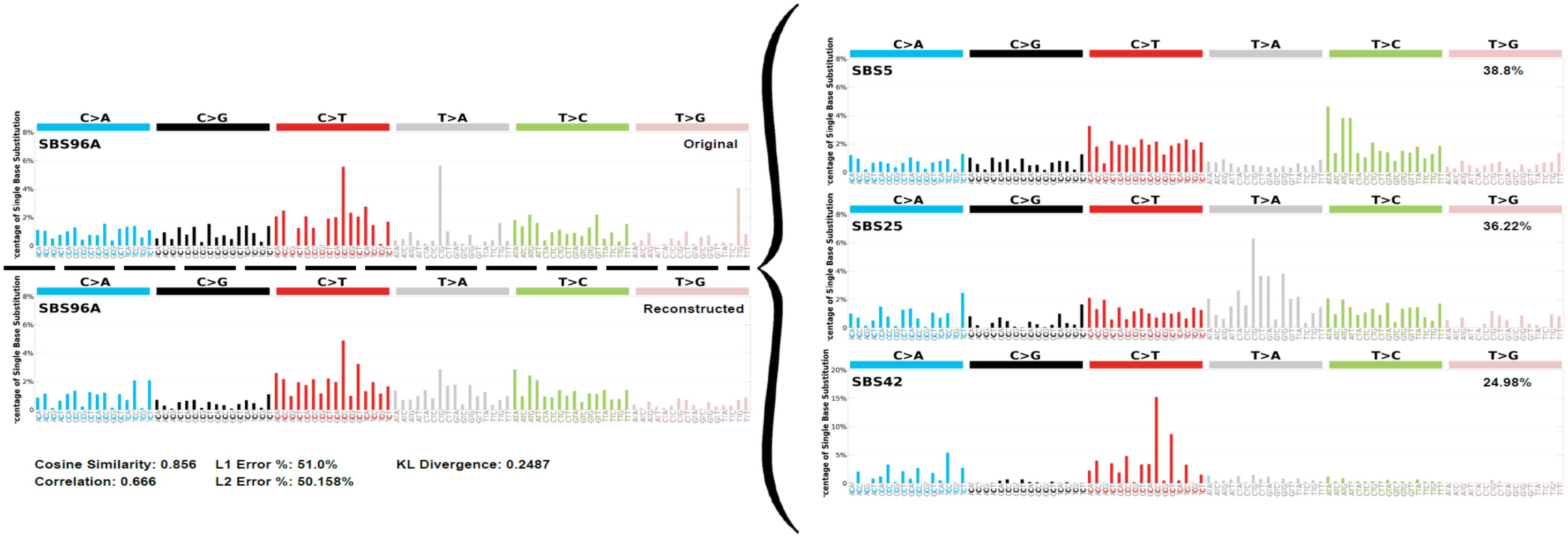

Supplementary Figure 13. Decomposition plots of mutational signature analysis with TCGA samples (n=80).

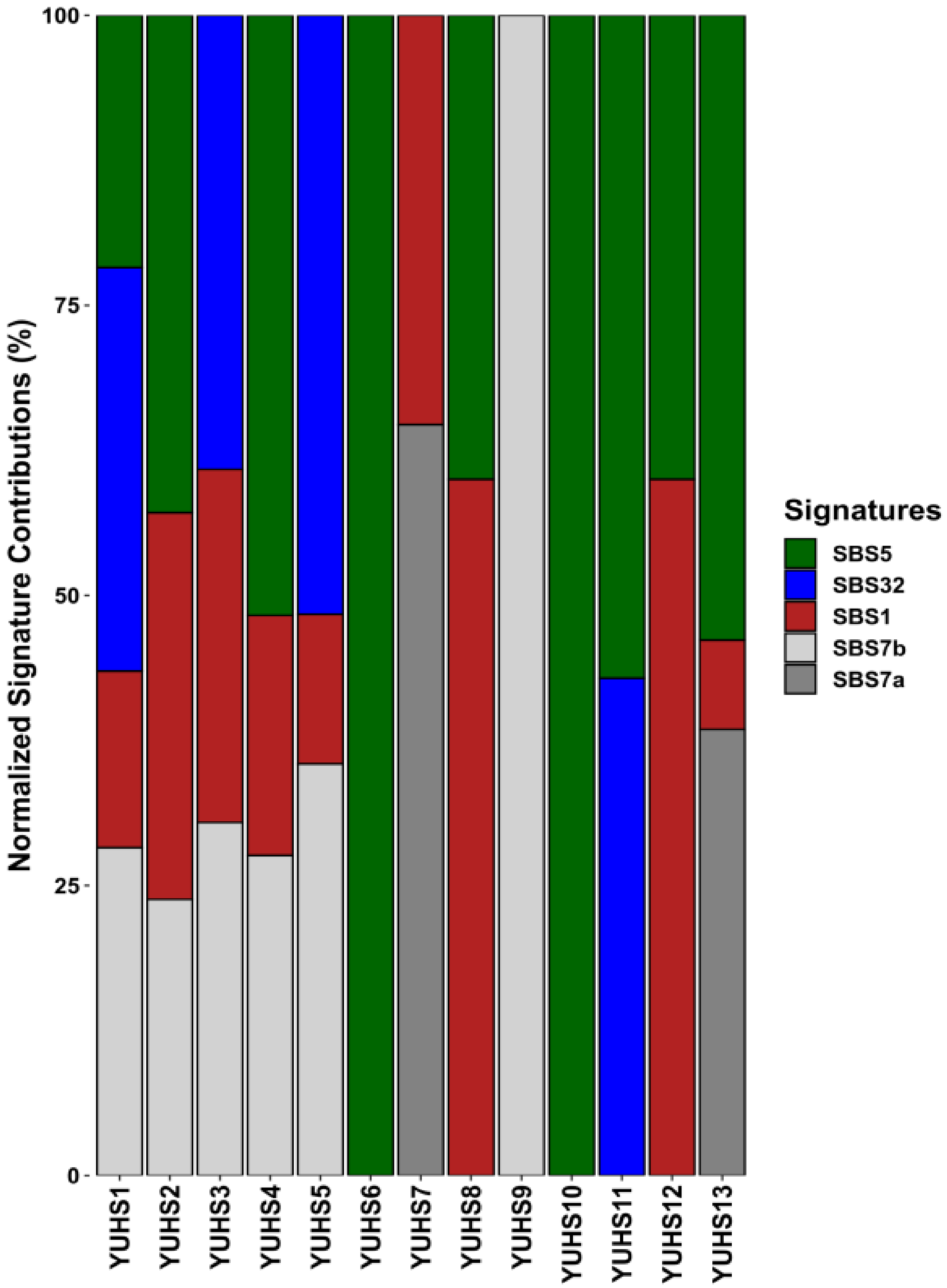

Supplementary Figure 14. Normalized mutational contribution of signatures per YUHS samples (n=13).
